# Supplementary material for: Are carbon emissions trading and green financial instruments synergistic? -Comprehensive quantitative research based on content analysis
Source: PLoS One. 2024 Mar 7;19(3):e0298601. doi: 10.1371/journal.pone.0298601 (PMC10919601; doi:10.1371/journal.pone.0298601)
Supplement: S1 Data — (ZIP) [file pone.0298601.s001.zip › Data/地级市Z_F工作报告.zip!/╔┬╬≈/╙▄┴╓╩╨/└┤╫╘╣1⁄2╓┌║┼í╛┬φ┐╦╩2╛▌═°í┐_2019.docx]

各位代表：

现在，我代表市政府向大会报告工作，请予审议，并请市政协委员和其他列席人员提出意见。

一、2018年工作回顾

2018年是全面贯彻党的十九大精神开局之年，也是榆林发展进程中极不平凡的一年。面对各种风险挑战和繁重发展任务，我们坚持以习近平新时代中国特色社会主义思想为指导，在市委的坚强领导下，树牢“四个意识”，坚定“四个自信”，坚决做到“两个维护”，紧扣追赶超越和“五个扎实”要求，坚持稳中求进工作总基调，围绕加快实现“三大目标”、发挥“两个更大”作用，聚焦转型升级、高质量发展，统筹推进稳增长、促改革、调结构、惠民生、防风险各项工作，较好地完成了市四届人大五次会议确定的各项任务。

——主要经济指标创“十三五”以来最好水平。实现生产总值3848.62亿元，增长9.0％，经济总量跃居西部第六、呼包鄂榆城市群第一，追赶超越取得重大进展；全社会固定资产投资增长8.5％；完成地方财政收入389.8亿元，增长24.5％；实现社会消费品零售总额514.11亿元，增长10.1％；城镇居民和农村居民人均可支配收入达到31317元和12034元，增长8.2％和9.1％。

——高端能化基地建设驶入快车道。确立“12363”煤化工产业高端化发展新战略，总投资超过2000亿元的重大转化项目加快推进，一大批填补产业空白的精细化工项目落地建设，“煤头化尾”全产业链竞争优势加速构筑。创造两项世界第一的榆能横山、陕能赵石畔电厂并网发电，陕北至湖北电力通道获批，总装机796万千瓦的外送电源点项目加快推进，电力项目建设取得历史性进展。

——一批事关全局和长远的大事要事取得重大突破。狠抓十大战略问题研究成果物化和27项阶段性重大事项，干部群众翘首以盼的延榆高铁即将开工，呼吁多年的煤炭资源税税率由6％提高到9％，事关长远的黄河东线马镇引水工程可研通过审查，榆阳机场开辟国际航线，一批打基础、利长远的重大工作正在加速破题，高质量发展的战略支撑不断增强。

——优化发展环境打了一个翻身仗。铁腕整顿营商环境、重拳整治舆论环境、下茬修复金融生态，营商环境排名由全省倒数第1提高到2018年上半年的第5位，在全国262个地级市综合信用排名中由倒数第3跃升到第86位，进位幅度全国第一，榆林重信守诺的人文传统正在回归。

——干部干事创业精气神有了新提升。持之以恒正风肃纪，认真做好以案促改，扎实开展“狠抓落实年”活动，树立新的发展观、资源观，扭转“形势一好就放松抓经济、煤价一涨就忽视调结构”思维定势和“工程冬眠、干部冬闲”工作怪圈，党员干部上下同欲、务实担当的氛围日趋浓厚。

一年来，我们主要抓了以下六方面工作：（一）全力保持经济稳中向好。认真落实中省宏观调控政策，研究制定推动工业高质量发展等系列政策措施，聚焦要素保障、市场需求等关键环节强化运行调度，严格落实各类奖补措施和清费降税政策，全年减免企业税额185.1亿元，新培育“五上”企业261户，规上工业直报企业利润总额居全省地级市首位。深化供给侧结构性改革，关闭落后煤炭产能135万吨，淘汰落后煤电产能18万千瓦，建立防止“地条钢”死灰复燃、严防严控“电解铝”长效机制，全市规上工业企业技改投资完成102亿元，增长5.3％。开展重大项目攻坚年活动，重点项目投资首次突破1000亿元，储备项目投资规模达到6077亿元，扭转了投资增长乏力、项目储备不足的被动局面。成立市招商委员会，出台12个招商引资优惠政策，山东信发循环经济产业园、深圳三科农商智慧城等一批骨干项目落地，全年招商引资实际到位资金881.37亿元，增长25.4％。

（二）坚决打好三大攻坚战。将防控金融风险摆在突出位置，出台违法失信联合惩戒实施意见，扩大信用报告使用范围，开展公职人员不良贷款专项治理，银行业不良贷款率由10.05％下降到4.78％；妥善化解了一批政府性债务风险点，区域金融生态大幅改善。深入开展安全生产“三项攻坚”和食药安全“雷霆行动”，全年未发生重特大安全生产事故和较大食品药械安全事件。扎实推进脱贫攻坚，全年扶贫领域投入资金90.9亿元，认真落实“八个一批”政策措施，11.69万人脱贫、432个贫困村退出，绥德、米脂、吴堡3个计划摘帽县完成市级核查。加强生态文明建设，狠抓中省环保督察反馈问题整改，扎实推进铁腕治污“14＋3”攻坚行动，城区环境空气质量优良率达到80.4％，15个国省控断面水质全部达标；国家森林城市创建有序推进，完成营造林面积74.5万亩，生态环境持续优化。

（三）加快构建现代产业体系。深入实施创新驱动战略，甲醇燃料技术装备行业创新中心等一批创新平台落户榆林，新增高新技术企业19户，高新技术企业产值突破300亿元。完成千亿元新能源和多晶硅规划编制，新能源新增装机53万千瓦，总装机达到723万千瓦，战略性新兴产业增加值增长11.4％。出台支持现代农业发展“16条”，设立6亿元农业产业发展基金，新增山地苹果11.9万亩，引进陕果集团等5户国内百强农业龙头企业，粮食总产量达266万吨，创历史新高。成功申报全国流通领域现代供应链体系建设试点城市，榆阳象道等一批现代物流园区加快建设，绥德无车承运试点投入运营，乡镇快递网点覆盖率和行政村直接通邮率均达100％，现代物流短板加速弥补。出台扩大旅游文化体育等领域消费的实施意见，扎实推进骨干景区建设，举办“陕北榆林过大年”“清爽榆林”等特色活动，全年接待游客和旅游综合收入分别增长34％和33％。积极发展电子商务、健康养老、信息服务等新业态，服务业增加值增速连续三年居全省第一。

（四）全面深化改革开放。深入推进“放管服”改革，544项行政许可和公共服务事项进驻市政务服务中心，开展“减证便民”专项行动，开通12345便民热线，43项收费目录清单、154项“最多跑一次”事项向社会公布，全年新增市场主体4.3万户，同比增长20.3％。完成国企“三供一业”改革。推开园区改革，产业园区数量由44个整合到23个。推进涉煤领域改革，煤炭资源转化引导基金首轮募资40亿元，煤炭矿业权清理整顿取得积极进展。推进电力体制改革，全年完成电力直接交易100.8亿度，增长43％。深化财税金融改革，财政预算绩效管理和“零基”预算制度全面推行，榆林农商行加快组建。落实农村土地“三权分置”改革，完成行政村清产核资，农村产权交易中心实现县域全覆盖。持续扩大对外开放，高质量举办第十三届榆林国际煤博会；榆阳机场扩建飞行区建成投用，年旅客吞吐量达到209万人次，居西北支线机场第一；扬榆协作成效显著，呼包鄂榆城市群合作日益紧密；全年实际利用外资8762万美元，增长25％，榆林的开放之路越走越宽。

（五）统筹城乡区域协调发展。完成第五版城市总规纲要、中心城区综合交通规划和古城保护修建性详规编制。科创新城启动建设，“四馆两中心”全面开工，榆溪河生态长廊二期北段等一批城市公园建成开放，红山路拓宽改造等52个市政项目扎实推进，万达广场建成投运，中心城区房地产项目历史遗留问题加快解决，国家文明城市、园林城市等创建工作有序推进，城市功能和品质持续提升。出台加快全市城镇化发展实施意见，吴定高速及242国道绥德过境线等4条国省公路建成通车，绥延高速完成投资34.5亿元，定边供水提升改建控制性工程开工建设；出台支持大柳塔镇级小城市综合改革试验区转型发展意见，横山波罗等3个省级文化旅游名镇、神木大保当等3个省考重点镇完成投资26.2亿元。完成市县两级乡村振兴战略规划编制，安排市级专项资金10亿元，首批204个乡村振兴示范村建设稳步推进，1013个重点村人居环境整治取得阶段性成果，城乡面貌发生新变化。

（六）倾心尽力办好民生实事。坚持发展成果由人民共享，全年民生支出525亿元，占财政总支出的80.9％。优先发展教育事业，出台深化教育综合改革意见，教师工资提高15％用于绩效考核；引进北师大、陕师大等优质教育资源；中心城区23个校建项目完成投资2.15亿元，普惠性幼儿园覆盖率达到86％，基本消除超大班额；去年，我们的老师和孩子们都很给力，全市高考二本上线率达到45％，创历史最好水平。深入推进健康榆林建设，与省内10所知名医院合作建立12个专科联盟，市精神卫生中心投入使用。稳步提升社会保障水平，城镇新增就业2.8万人，转移农村劳动力就业42.3万人；普通人群和贫困人口大病医保最高支付额分别提高到45万元和70万元；建成全省首个留守儿童和困境儿童关爱保护中心。开展医养融合发展试点，新建农村幸福院423个、覆盖率提高到42％。实现县级数字影院和乡村两级公共文化服务站室全覆盖，陕北民歌博物馆建成开放，“榆林古城六楼民间文化展演”列为国家级文化服务示范项目。省十六运取得奖牌榜第二的优异成绩，成功申办省十七运，举办首届榆林国际马拉松赛。扎实推进“平安榆林”建设和扫黑除恶专项斗争，依法侦办了一批影响恶劣的涉黑涉恶和新闻敲诈勒索案件，社会治安防控体系更加完善，公众安全感和满意度持续提升。

过去的一年，我们大力推进政府自身建设，坚决落实全面从严治党要求，认真做好省委巡视反馈意见整改，严格落实中央八项规定及其实施细则精神和省委、市委实施办法。加强法治政府建设，自觉接受人大法律和工作监督、政协民主监督和社会监督，人大代表建议156件、政协委员提案376件全部办复。国防动员和双拥共建深入开展，民族宗教、司法、新闻广电、市场监管、统计、人民防空、气象地震、外事侨务、老龄、残疾人、史志档案、供销等工作都取得了新成绩、新进步。

各位代表，回望过去一年，我们革故鼎新、兴利除弊，全力廓清制约榆林高质量发展的各类顽瘴痼疾，朝着全面建成小康社会目标迈出坚实步伐。成绩来之不易，这是习近平新时代中国特色社会主义思想科学指引的结果，是省委、省政府和市委坚强领导的结果，是市人大和市政协监督支持的结果，是全市干部群众撸起袖子干出来的、挥洒汗水拼出来的。在此，我代表市政府，向全市人民，向人大代表、政协委员，向各民主党派、工商联、无党派人士和各人民团体，向驻榆解放军、武警部队官兵，向中省驻榆企业和国内外客商，向所有关心支持榆林改革发展的社会各界人士，表示衷心的感谢并致以崇高的敬意！

我们也清醒地认识到，我市发展不平衡不充分的深层次问题依然突出：经济持续增长基础不牢，产业层次和发展质量不高，转型升级步伐不快；改革任务繁重，创新能力不强，对外开放水平不高，人才和金融支撑不足，改善发展环境任重道远；脱贫攻坚任务艰巨，生态环保、安全生产压力加大，金融风险仍然存在；民生事业与群众期盼还有差距；干部作风与形势任务要求还不适应，形式主义、官僚主义还比较突出。这些都是榆林全面小康路上躲不开、绕不过的坎，是我们必须战胜的拦路虎。我们将始终保持忧患意识和昂扬斗志，迎难而上、砥砺前行，以抓铁有痕、踏石留印的作风，把这些问题解决好，开创新时代榆林转型升级、高质量发展新局面！

二、2019年重点工作

今年是新中国成立70周年，是全面建成小康社会的关键之年，做好今年工作意义重大。当前，世界面临百年未有之大变局，我国经济运行稳中有变、变中有忧。中央“六稳”政策全面发力，积极的财政政策和稳健的货币政策加力提效，基础设施补短板、更大规模减税降费、扩大债券规模等稳增长政策加快落地，为经济持续健康发展注入了新动力。特别是地缘政治冲突刺激全球能源价格高位运行，榆林14项国家战略规划覆盖的叠加效应加快释放，现代煤化工在全球能源版图中的地位持续提升，一系列补短板、管长远的大事要事破题推进，榆林正处于转型升级、高质量发展的重大机遇期和历史窗口期。我们将牢记使命，乘势而上，坚定不移抓机遇、用机遇，充分调动各方面积极因素，一锤接着一锤敲，一张蓝图干到底，努力建设一个更加富裕文明和谐美丽的现代化新榆林！

按照市委四届六次全会安排，2019年政府工作的总体思路是：坚持以习近平新时代中国特色社会主义思想为指导，全面贯彻党的十九大和十九届二中、三中全会、中央经济工作会议、省委十三届四次全会和省“两会”精神，坚持稳中求进工作总基调，坚持新发展理念，坚持以供给侧结构性改革为主线，坚持深化市场化改革、扩大高水平开放，紧扣追赶超越和“五个扎实”要求，围绕实现“三大目标”、发挥“两个更大”作用，聚焦转型升级、高质量发展，统筹推进稳增长、促改革、调结构、惠民生、防风险各项工作，进一步稳就业、稳金融、稳外贸、稳外资、稳投资、稳预期，提振市场信心，增强人民群众获得感、幸福感、安全感，保持经济持续健康发展和社会大局稳定，以优异成绩向新中国成立70周年献礼！

主要预期目标是：生产总值增长7.5％左右；规上工业增加值增长6％左右；地方财政收入增长8％以上，力争实现430亿元；全社会固定资产投资力争达到2000亿元；社会消费品零售总额增长10％左右，城乡居民人均可支配收入分别增长8％和9％以上。围绕上述目标，重点做好七方面工作。

（一）聚焦关键环节精准发力，推动经济持续健康发展

做强做优实体经济。适时出台稳增长政策措施，抓好重点地区、重点行业、重点企业运行调度；开展千户企业大调研活动，推动企业复产复工、释放产能，确保榆神煤基混合芳烃等35个新增产能项目达产达效、大梁湾等12处在建煤矿转产投运、大海则煤矿开工建设、巴拉素等2处煤矿取得核准；规范并加大油气勘探开发，保持油气产量稳定增长。提升要素保障水平，严格落实中省减负降本一揽子政策措施，实行小微企业“零成本”接电，全年直供电交易规模增长20％以上，载货类车辆高速通行费下降9％，支持陕能投、榆能集团、北元化工在主板上市。把支持民营经济发展摆在更加突出的位置，严格落实支持民营经济高质量发展意见，开展清理拖欠企业账款专项行动；落实融资担保“十条措施”，引导金融机构扩大信贷投放，支持企业发行债券，着力破解融资难题；实施“育小扶中抓大”工程，落实民营企业“小升规”奖补政策，全年“五上企业”数量增长20％以上；继续发布民营企业行业榜和百强榜，扩大优秀高校毕业生到民企工作选聘规模；鼓励各级领导干部坦荡真诚与企业家交往，理直气壮支持企业发展，构建“亲”“清”政商关系。

积极扩大有效投资。继续开展“重大项目攻坚年”活动，严格领导包抓、挂图作战、按月调度、季度点评推进机制，确保国华锦界电厂三期等131个续建项目3月底前全部复工、未来能源一期后续工程等170个新建项目6月底前实质性开工，力争华能段寨煤电一体化等96个前期项目年内具备开工条件，迅速形成大干快上的项目建设新热潮。抢抓国家新时代推进西部大开发、深入实施东西部扶贫协作等政策机遇，围绕全市重大战略布局和十大战略问题研究，谋划储备一批具有引领性、支撑性的重大项目，并争取列入中省规划。夯实县市区、园区招商主体责任，围绕我市重点产业，科学绘制产业图谱和招商地图，广泛宣传招商引资优惠政策，主攻投资体量大、科技含量高、带动能力强的项目，力争全年招商引资到位资金突破1000亿元。

持续优化营商环境。深化“放管服”改革，加快启用市级政务服务“一张网”，政务服务事项网办率达到70％以上，重点领域和高频事项基本实现“一网、一门、一次”目标；巩固扩大“证照分离”改革成果，破解“准入不准营”难题；开展行政审批事项颗粒化梳理工作，推行综合受理、容缺受理、联办通办、证照包邮等服务新模式，打通服务群众“最后一公里”；推进“双随机、一公开”监管全覆盖，实现事中事后监管制度化、规范化。抓好优化营商环境“十大行动”，大力推进工程建设项目审批制度改革，实质性启动榆神、榆横工业区企业投资项目承诺制改革；采取第三方评估、公开排名等方式，推动全市营商环境大提升。建立健全信用信息汇集、核查机制，继续扩大信用报告使用范围，定期发布诚信“红黑榜”“灰名单”，严厉打击非法集资、金融诈骗、恶意逃废债等违法行为，确保我市在全国的信用排名持续提升。

（二）集中力量攻坚克难，坚决打好三大攻坚战

有效防范化解重大风险。落实总体国家安全观，增强忧患意识，切实防范各类风险挑战。继续抓好金融风险防控，坚决遏制不良贷款增量，积极处置化解存量，确保全市金融机构不良贷款率控制在4％以内；加大政府债券争取力度，妥善化解隐性债务。突出安全领域风险管控，深刻汲取近年来特别是神木1.12矿难惨痛教训，以对党负责、对历史负责、对人民负责的态度，坚决打赢煤矿安全生产和生态环境保护两场硬仗，同步开展覆盖社会全行业领域的安全隐患大排查大整治，推动安全形势持续稳定好转。深入实施食品放心工程，全面推行量化分级动态评级，扎实开展城乡结合部、学校食堂、小餐饮等重点领域食品安全问题专项整治；加大对制售假劣药品、虚假宣传等违法行为的打击力度，坚决守住人民群众生命健康安全防线。

决战决胜脱贫攻坚。把脱贫攻坚作为重大政治任务，严格“两不愁三保障”标准，一鼓作气、尽锐出战，确保3.2万贫困人口脱贫、剩余的134个贫困村退出，佳县、清涧和子洲三县摘帽。继续加大财政投入，推进山地苹果、设施农业、特色养殖等产业发展，做好就业、生态、教育、健康、金融和兜底保障等扶贫工作，完成“两房”建设目标任务。实施“企村共建”行动，提升扬榆扶贫协作质量水平，巩固“三位一体”大扶贫格局。坚持抓脱贫和防返贫并重，健全稳定脱贫长效机制，确保脱贫不脱政策，脱贫不脱帮扶。坚持扶志和扶智相结合，充分激发贫困群众内生动力和自我发展能力，确保全面小康路上不落一户一人。

持续发力治理生态环境。认真落实习近平生态文明思想，坚决扛实生态建设政治责任，深入实施铁腕治污“14＋3”攻坚行动，着力打好蓝天、碧水、净土、青山四大保卫战。开展新一轮蓝天保卫三年行动计划，加大城区散煤禁烧治理力度，完成工业挥发性有机物深度治理、工业燃煤锅炉超低排放改造和储煤场环保改造三项重点任务。严格落实河长制、湖长制，扎实推进无定河全线综合治理，启动城市污水处理提标改造，彻底消除榆阳河、沙河、榆溪河黑臭水体，启动矿井疏干水综合利用示范工程建设。强化土壤质量检测和污染管控，持续推进畜禽养殖废弃物资源化利用，推行油气开采废弃物、工业固废集中达标处置，完成工业园区及重点镇“两场”建设改造。全面推进国土绿化，实施环榆百万亩生态圈、城镇绿化等十大林业重点工程，完成营造林70万亩，力争国家森林城市创建通过中省验收。以铁的决心，下硬茬彻底整治“明盘”项目，全面修复矿区生态环境。

（三）深化供给侧结构性改革，加快构建现代产业体系

推进能源化工基地高端化。出台高端能化基地建设“1＋3”规划，启动编制重点园区“五个一体化”发展规划，建好榆横终端产品加工园、榆神精细化工产业园等4个“园中园”，加快构建特色鲜明、错位发展、优势互补的产业园区新格局。落实《推进煤化工产业高端化发展若干政策实施细则》，出台平价煤资源配置管理办法，启动运营煤炭资源转化引导基金，设立现代煤化工等子基金，推动陕煤煤制化工新材料等一批重大项目落地。坚持双周实战推进例会制度，推动中煤二期、未来二期、神华二阶段等11个重大转化项目开工建设，做好榆能中科400万吨煤制油等8个项目的前期工作；开工建设陕北至湖北输电工程及3个配套电源点项目，启动陕北至关中第三通道和陕北至浙江输电通道前期工作，推开高端能化基地建设新局面。

加快传统产业升级改造。启动传统产业升级改造三年行动计划，通过重组整合、重大技术攻关、产业基金引导、财政资金奖补、环保安全倒逼等方式，促进传统产业焕发生机活力。抓好府谷东方希望480万吨氧化铝、神木利臻5万吨高端型材等骨干项目建设，推进府谷金属镁工程技术研究中心和质量检测检验中心建设，提升铝镁产业竞争力。加快兰炭产业绿色升级改造，淘汰置换一批污染严重、整改无望的落后产能，推动煤炭分质分级清洁高效利用。大力发展工业互联网，组织实施“企业上云”行动，创建一批示范数字化车间和智能工厂。抓住中东部轻纺产业转移历史机遇，加快突破服装设计、品牌营销等关键环节，提升羊毛防寒服全产业链发展水平。

打造千亿现代服务业集群。做强做大旅游文化产业，编制全域旅游发展总体规划，加快靖边波浪谷一期、扶苏文化苑二期等文旅项目建设，推进佳县白云山等5A级景区创建；完善旅游配套设施建设，培育发展文化体验游、自驾房车游等新业态，打造高品质旅游“景点群”和“线路套餐”；深入挖掘石峁、统万城等世界级旅游资源潜力，打造榆林旅游文化国际名片。大力发展枢纽经济、门户经济、流动经济，推进神木西站、靖边集运站等现代物流园区建设，发展无车承运、多式联运、智慧物流等新业态，推动京东物流仓储中心落地，打造全国综合交通枢纽城市和陕甘宁蒙晋交界快递物流中心。依托靖边无人机试验测试基地，大力发展集无人机、无人车、物联网、5G通信、人工智能于一体的综合无人系统，打造国内领先的智能无人产业聚集区和科技旅游体验区。加快发展工业设计、设备维修、融资租赁、信息技术等生产性服务业，推动制造业和服务业深度融合。

（四）坚持农业农村优先发展，大力实施乡村振兴战略

着力构建现代农业产业体系。培育壮大特色优势农业，建设苹果、蔬菜、马铃薯、羊子4个百亿级产业基地，大力发展小杂粮、核桃、海红果等区域特色种植业，新增优质山地苹果15万亩、中药材5万亩，改造红枣低产园10万亩。抓好非洲猪瘟等重大动物疫病防控，保障畜产品安全稳定供给。实施现代农业产业园、农业科技示范园提质增效工程，推进榆阳区国家农村产业融合发展示范园建设。加快绥德陕果集团产销服务中心、定边马铃薯主食化二期、米脂小米酵素生态园、子洲黄芪综合开发等项目建设，推动一二三产融合发展。加快果蔬物流冷链综合体建设，支持电商农业、订单农业、休闲农业发展，打造一批种养一体、农旅融合的田园综合体。坚持“农地姓农”，抓好“大棚房”整治。大力实施农产品质量安全和品牌建设，加强“三品一标”认证工作，培育整合以“榆林尚农”冠名的拳头品牌，让更多榆林绿色农产品走向全国人民的餐桌。

积极打造生态宜居美丽乡村。切实抓好乡村振兴示范村建设，在巩固提升现有204个村的基础上，再启动100个，精心打造一批乡村振兴示范样板。深入推进农村人居环境整治，学习借鉴浙江“千村示范、万村整治”经验，加大拆违治乱、植树增绿力度，完成1000个村的生活垃圾治理，让广大农民生活得更有尊严。推动农村基础设施提档升级，创建“四好农村路”示范里程1000公里，加快农村电网升级改造，解决好群众安全饮水问题，不断夯实乡村振兴基础。健全城乡融合发展体制机制，鼓励引导市民下乡、能人回乡、企业兴乡，努力构建工农互促、城乡互补的新型工农城乡关系。

持续深化农业农村综合改革。深化农村土地制度改革，落实承包地“三权分置”配套改革政策，因地制宜推广“一户一块田”、土地托管等模式，有序发展适度规模经营。稳慎推进农村宅基地改革。深化农村集体产权制度改革，总结提升榆阳区赵家峁“三变”改革经验，继续实施“百村试点千村推进”工程，巩固提升现有209个试点村改革成果，逐步消除集体经济空白村。建立市县乡村四级“三资”管理信息平台，实现农村土地承包经营纠纷调解仲裁机构县域全覆盖。深化农村经营体制改革，加大职业农民、家庭农场、农民合作社和产业化联合体等新型经营主体培育力度，着力破解小生产与大市场之间的矛盾。

（五）加快建设最具影响力城市，打造靓丽宜居美好家园

全面提升中心城市功能品质。开展城市品质三年提升行动，严格市域空间一体管控，启动国土空间总体规划编制，同步推进第五版城市总规和科创新城控规编制；做好第三次全国国土调查，彻底解决长期存在的地类认定不一致等问题。高水平推进科创新城建设，启动榆林学院新校区建设，铺开总投资26亿元的基础设施工程；切实抓好古城开发保护，完成东城墙修复，实施古城亮化工程，优化古城业态布局；加快推进主城区重点项目建设，完成“四馆两中心”主体工程，启动凌霄广场重建、汽车南站迁建，继续抓好棚户区改造。全面推进城市严管、细管和智管，扎实推进各类创建活动，集中开展交通秩序、广告牌匾、环境卫生等专项治理；深化智慧城市建设，建立统一的大数据、政务云平台，上线一批便民利企的智慧创新应用；坚持“房住不炒”理念，适时出台调控政策，推动房地产市场健康发展；继续抓好中心城区房地产项目历史遗留问题化解工作，整顿规范住宅小区物业管理，推进户表改造，努力建设更高品质的和谐宜居城市。

统筹推动区域协调发展。按照北部转型发展、南部绿色发展的总体部署，建立更加有效的区域协调发展新机制，出台引导首位产业发展专门政策，支持北部县市区发展能化终端产品、轻纺、铝镁合金等转型产业，加大对南部县文化旅游、中药材种植、农产品深加工等特色产业扶持力度，推进沿黄生态经济带建设，加快形成各具特色、优势互补、协同并进的县域经济发展新格局。继续抓好绥德、子洲灾后重建，全力推进污水管网、学校、医院等重点项目。深入实施“一县一镇”示范工程，切实抓好省级文化旅游名镇和省考重点镇建设，集中力量打造一批特色鲜明的重点镇。

强力推进重大基础设施建设。落实加快城镇化发展实施意见，集中力量补短板、强弱项，不断提升城镇综合承载力和辐射带动能力。机场方面，完成榆阳机场扩建航站楼主体工程及相关配套设施，开工建设府谷支线机场和靖边通用机场，加快定边支线机场前期工作。铁路方面，开工建设延榆高铁，建成靖神铁路和蒙华铁路榆林段。公路方面，抓好清子高速、307国道靖定段等国省干线公路建设，确保绥延高速年内建成通车。水利方面，开工建设黄河东线马镇引水工程和绥米子三县供水项目，力争定边供水提升改建工程建成通水。通讯设施方面，扩大4G网络农村覆盖范围，启动5G网络建设规划编制，着力打造“无线榆林”。

（六）全面推进改革开放创新，激发加快发展新动能

纵深推进重点领域改革。有序推进市县机构改革，完成职责调整和工作衔接。加快投融资体制改革，制定政府投资项目管理办法和三年滚动计划。深化国资国企改革，理顺市属国企监管体制机制，完善企业法人治理结构，加强党组织和“三会一层”建设，全面推进榆能集团内部改革和战略重组。深化产业园区改革，完成园区整合、管委会机构撤并任务，推开园区行政审批制度、人事薪酬制度、管理体制、运营机制改革。有序推进涉煤领域改革，分批次开展煤炭矿业权清理整顿，加快组建榆林能化交易中心，推动国有企业煤炭交易全部实现网上运行，提升“榆林煤”价格指数影响力。全面推进公共资源交易制度改革，确保公共资源交易“线下无交易、线上全留痕”。推进电力体制改革，协调理顺网间输配电价，开展靖边能化园区、府谷高新区、榆横工业区增量配电试点改革。

全方位扩大对外开放。出台全面扩大对外开放政策，支持企业在境外设立海外仓、商品展示中心，依托包头、西安中欧班列，扩大与“一带一路”沿线地区经贸合作。积极申报一类航空口岸，开通直飞日本、韩国等国际航线，推动B型保税物流中心建设，简化检验检疫和通关流程，积极培育引进外贸综合服务企业，扩大金属镁、化工产品、特色农产品出口规模。办好榆林国际煤博会、镇北台论坛等展会论坛，借力国内外顶级会展平台推介“榆林好产品”。落实呼包鄂榆城市群发展规划，启动乌兰木伦—大柳塔蒙陕合作试验区建设；加强与京津冀、长三角、珠三角等经济圈联系对接，落实扬榆经济合作框架协议，在产业转移、招才引智等方面深化合作。

深入实施创新驱动战略。扎实推进国家创新型城市试点建设，落实支持科技创新若干政策，发挥好科技成果引导转化基金作用，健全科技成果转移转化直通机制，推动更多创新活动在榆结出硕果。积极推动与西安科技大学共建煤炭绿色安全开采国家重点实验室，依托榆林学院创建开放型大学，新建一批市级工程技术研发中心、重点实验室和中试基地，力争技术合同成交额增长20％以上。大力实施“卡脖子”关键核心技术攻坚行动，主动跟进能源领域颠覆性技术和新兴产业前沿技术，在粉煤热解、金属镁、新材料等关键领域寻求突破，全社会研究开发经费投入强度提高到0.25％以上。加快出台更有吸引力的“招才引智”政策，用有温度的政策汇聚人才、留住人才。

（七）坚守初心使命，持续保障和改善民生

办好人民满意教育。推动义务教育均衡发展，确保绥德国家义务教育发展基本均衡县创建通过验收。深入实施学前教育三年行动计划，开展城镇小区配套幼儿园治理，推开公办园差额收费、民办园差异奖补政策，普惠性幼儿园占比提高到87％以上。加快推进中心城区校建工程，确保北师大榆林附属学校小学部和幼儿园年内开工、中学部秋季开始招生。实施“百千万”名优教师培育工程，全面加强师德师风建设和学生文明礼仪教育。强化校外培训机构监管，切实减轻学生课业负担。推动职业教育多元办学、校企合作和产教融合，提升特殊教育、继续教育办学水平。我们一定要把榆林教育的质量提上来，兑现“努力不让孩子为求学远赴他乡、家长为抚育子女两地奔波”的诺言。

深入推进健康榆林建设。开工市中医院迁建和市疾控中心综合服务楼项目，新建40个标准化乡镇卫生院和200个规范化村卫生室，促进优质医疗资源向基层下沉。深化专科联盟内涵建设，集中培育12个省级、16个市级重点专科，积极引进高层次医疗人才和专家团队，巩固提升95％的市域就诊率；启动疾控重点学科建设，扎实推动8类健康细胞示范建设工作，推进疫苗流通使用专项治理，筑牢健康榆林基础。深化医疗卫生体制改革，实行全员编制岗位总额管理，落实总会计师和总药师选派制度，推动市内医疗机构检查结果共享互认，让百姓看病省心省力省钱。

不断提升社会保障水平。实施普惠性支持政策，加大对农民工返乡创业政策扶持力度，扎实做好高校毕业生、城镇就业困难人员、农村转移劳动力等重点人群就业工作，确保零就业家庭动态清零。加快推进社会保障卡应用，实施全民参保计划，推进重点行业、重点人群的社会保险扩面工作。健全城乡养老服务体系，完善农村互助幸福院可持续运行机制，落实医养结合实施意见和引导社会资本兴办养老机构优惠政策，新建一批社区日间照料中心和农村互助幸福院。健全留守儿童和困境儿童“五位一体”救助保障制度，有效落实扶残助残各项政策，让弱势群体感受到党和政府的关爱和温暖。做好退役军人安置、管理和保障工作，加强退役军人政治思想教育和就业创业技能培训，维护军人军属合法权益，增进军政、军民团结。

大力繁荣文化体育事业。深化全国文明城市创建，弘扬践行社会主义核心价值观，持续开展“厚德榆林”“文明家庭”等评选活动，力争在全国文明城市年度测评中进入前30位。切实加大对文物古迹、红色革命旧址、非物质文化遗产等重要文化载体的保护开发，确保国家级陕北文化生态保护实验区通过验收。加快推进县城图书馆、文化馆和社区农村综合文化服务中心建设，提升公共文化服务共建共享水平。做好省十七运筹备工作，加快职业技术学院体育馆等场馆建设，实施一批便捷实用的运动公园、健身步道等体育惠民工程；持续扩大榆林国际马拉松、吴堡黄河漂流等赛事影响力，以品牌赛事引领全民健身新风尚。

打造共建共治共享社会治理格局。持续做好城市社区达标改造和农村社区建设，加大各类社会组织培育力度，提高社区服务能力和水平。推进公共法律服务体系建设，抓好基层人民调解工作；开展领导干部包案接访活动，集中化解信访积案和复杂矛盾，依法打击各类非访行为。深入开展“平安榆林”建设，纵深推进扫黑除恶专项斗争，加大对极端暴力、黄赌毒、盗抢传销、电信诈骗等犯罪行为的防范打击力度，持续深化社会治安防控体系建设，加快“雪亮工程”建设进度，提升全市人民的安全感。支持工会、共青团、妇联、慈善等事业发展，做好民族宗教、人防、外事、史志档案、气象地震、供销等工作。

各位代表，民之所望，政之所向。去年我们进行了首次民生实事公开征集，共梳理确定了今年中心城区的十件民生实事：一是解决大班额问题，新建中小学、幼儿园16所，新增学位1万个以上。二是解决停车难问题，整治车辆乱停乱放，新增停车位1万个、非机动车停车位3000个。三是优化便民市场布局，新设置便民市场5个以上，因地制宜设置一批便民早市和夜市。四是改造老旧小区，启动灵秀街片区、保龄球馆北片区2个老旧小区示范改造。五是建设城市休闲公园，建成榆溪河生态长廊二期、大墩梁山体公园，新建至少6个社区公园。六是上线运行群众生活手机APP，实现一批行政审批和便民服务事项“指尖办”。七是推进城区“厕所革命”，优化公厕布点，新建至少35座公厕。八是优化公交线路和交通信号灯设置，根据群众实际需求新设、改造一批公交站点，实现公交一卡通省市互联互通。九是整治校园周边环境，试点推行幼儿园和小学“弹性放学”，新建一批校园门口人行天桥。十是推进过敏性鼻炎防治研究，建成4个空气花粉暴片监测网点。

各位代表，民生无小事，枝叶总关情。我们将满怀感情和责任，全力以赴把这些实事办成办好，年底对标交账。各县市区也将同步开展民生实事公开征集，着力解决群众关心关注的痛点难点问题，努力为全市人民带来更多实打实的获得感！

三、切实加强政府自身建设

新形势新任务对政府工作提出了新要求。我们将以刀刃向内的革新勇气，把全面从严治党要求落实到从严治政之中，加快政府职能转变，提升政府系统的执行力、战斗力和公信力，努力锻造政治忠诚、务实担当、清正廉洁的人民满意政府。

旗帜鲜明讲政治。树牢“四个意识”，坚定“四个自信”，坚决做到“两个维护”，在思想上政治上行动上同以习近平同志为核心的党中央保持高度一致。深刻汲取秦岭北麓西安境内违建别墅问题教训，扎实开展“讲政治、敢担当、改作风”专题教育和“不忘初心、牢记使命”主题教育，切实增强政治敏锐性和政治鉴别力，坚决整治不敬畏、不在乎、表态多、调门高、行动少、落实差等行为，推动中省和市委各项决策部署落地生根。

真抓实干敢担当。依法接受人大及其常委会监督，自觉接受政协民主监督，主动接受社会和舆论监督，加强法治政府建设，真正做到权力在阳光下运行，干部为人民服务。全面推行“一线工作法”和重大事项台账管理制度，精准落实市直部门和县市区工作责任。强化督查问责，实行定期督查调度和红黄牌制度，坚决整顿懒政怠政行为。大力倡导敢闯敢试、敢破敢立、敢抓敢管的担当精神，用好“三项机制”，激励广大干部担当奉献、干事创业。

驰而不息改作风。大兴学习调查研究之风，增强“八种本领”，锤炼“五个过硬”，推动以战略问题研究破解难题常态化。牢固树立过“紧日子”思想，严格财政资金预算绩效管理，开展“破难查漏”专项行动，确保一般性支出压减5％以上。认真落实“一岗双责”，严格遵守中央八项规定实施细则和省委、市委实施办法，严查“四风”变异问题，集中整治形式主义、官僚主义，以过硬的作风为转型升级、高质量发展保驾护航。

从严治党管干部。狠抓巡视整改和“以案促改”，严明党章党规党纪，以整改堵漏洞，以治标促治本，激浊扬清、革弊立新。紧盯资源开发、工程建设、国资监管等重点领域，切实加强审计监督和廉政风险防控，全面扎紧制度笼子。严格执行领导干部廉洁从政纪律底线规定，重拳出击，铁面执纪，让纪律和规矩成为不可触碰的底线，动真碰硬解决“管党治党不严、干部管理不严、社会治理不严”问题，全面修复净化政治生态。

各位代表！征程万里风正劲，重任千钧再扬鞭。让我们更加紧密地团结在以习近平同志为核心的党中央周围，高举习近平新时代中国特色社会主义思想伟大旗帜，在市委的坚强领导下，紧紧依靠380万榆林人民，以更大的格局、更大的视野、更大的胸怀，奋力推进“三大目标”建设，迈出追赶超越新步伐，为决胜全面小康、实现转型升级、高质量发展而努力奋斗！
